# Combining acoustic tracking and LiDAR to study bat flight behaviour in three-dimensional space

**DOI:** 10.1186/s40462-023-00387-0

**Published:** 2023-04-26

**Authors:** Claire Hermans, Jens C. Koblitz, Harm Bartholomeus, Peter Stilz, Marcel E. Visser, Kamiel Spoelstra

**Affiliations:** 1 Department of Animal Ecology, Netherlands Institute of Ecology (NIOO-KNAW), Wageningen, The Netherlands; 2 Max Planck Institute of Animal Behavior, Constance, Germany; 3 Laboratory of Geo-Information Science and Remote Sensing, Wageningen University & Research, Wageningen, The Netherlands; 4 Animal Physiology, Institute for Neurobiology, University of Tübingen, Tübingen, Germany

**Keywords:** Acoustic localisation, Bats, Flight behaviour, LiDAR, Microphone arrays, Terrestrial laser scanning

## Abstract

**Background:**

Habitat structure strongly influences niche differentiation, facilitates predator avoidance, and drives species-specific foraging strategies of bats. Vegetation structure is also a strong driver of echolocation call characteristics. The fine-scale assessment of how bats utilise such structures in their natural habitat is instrumental in understanding how habitat composition shapes flight- and acoustic behaviour. However, it is notoriously difficult to study their species-habitat relationship in situ.

**Methods:**

Here, we describe a methodology combining Light Detection and Ranging (LiDAR) to characterise three-dimensional vegetation structure and acoustic tracking to map bat behaviour. This makes it possible to study fine-scale use of habitat by bats, which is essential to understand spatial niche segregation in bats. Bats were acoustically tracked with microphone arrays and bat calls were classified to bat guild using automated identification. We did this in multiple LiDAR scanned vegetation plots in forest edge habitat. The datasets were spatially aligned to calculate the distance between bats’ positions and vegetation structures.

**Results:**

Our results are a proof of concept of combining LiDAR with acoustic tracking. Although it entails challenges with combining mass-volumes of fine-scale bat movements and vegetation information, we show the feasibility and potential of combining those two methods through two case studies. The first one shows stereotyped flight patterns of pipistrelles around tree trunks, while the second one presents the distance that bats keep to the vegetation in the presence of artificial light.

**Conclusion:**

By combining bat guild specific spatial behaviour with precise information on vegetation structure, the bat guild specific response to habitat characteristics can be studied in great detail. This opens up the possibility to address yet unanswered questions on bat behaviour, such as niche segregation or response to abiotic factors in interaction with natural vegetation. This combination of techniques can also pave the way for other applications linking movement patterns of other vocalizing animals and 3D space reconstruction.

**Supplementary Information:**

The online version contains supplementary material available at 10.1186/s40462-023-00387-0.

## Background

Vegetation structure is a key biotic factor that affects animal movement [1]. Vegetation influences, among others, prey-predator interactions as it offers shelter from predators [2, 3]. While some nocturnal species are night active to reduce predation risk [4, 5], they often make use of the vegetation cover to find additional shelter [6]. Bats in particular depend on nocturnal darkness [7, 8]. Even in darkness, only a limited number of species ventures out in relatively open space when foraging, and these are generally fast-flying and manoeuvrable species [9, 10]. Many other species stay in cluttered environments when foraging; these are typically slow-flying species [9, 10]. Therefore, vegetation structure strongly influences bat activity and niche segregation between bat guilds [11, 12]. Studying the effect of vegetation structure on the behaviour and ecology of bats is often done at the landscape level [13–16]. Detailed information on how bats from different bat guilds adjust their small-scale spatial behaviour to vegetation structure is highly important to understand species-habitat relationships in forest environment [11, 12, 17].

Bat guilds may not only be differentiated by their flight behaviour, but also by their echolocation calls [9, 18]. Bats rely on echolocation to navigate, avoid obstacles and locate prey [19, 20]. As bats adjust their calls to the task and environment they are faced with [12], the structure of natural vegetation is a strong driver of call characteristics. Generally, calls differ with distance to background objects, such as the ground and vegetation. Such changes include increasing bandwidth and shortening interval and call duration when approaching vegetation [21]. However, the transition zone between open- and edge-space calls has been studied with limited spatial resolution [22, 23]. Precise measures of call parameter adjustment in response to the background can only be estimated by combining the position of a bat at the time of the call emission with the distance to the vegetation and the ground.

Knowing the precise positions of bats relative to vegetation structures is essential to understand the response of bats to different environmental factors. For example, there is accumulating evidence that bats’ behaviour is affected by ambient light. In line with nocturnality in general, the response of bats to low light levels at night is widely recognized as related to predator avoidance [4, 5]. This hypothesis is supported by the fact that slow-flying bats emerge later from their roosts compared to fast and agile bats [8]. Higher ambient light levels may cause bats to fly closer to vegetation structures in order to be less conspicuous to potential predators. This may reduce the possibility to observe bats and indeed, several studies reported lower activity of bats with moonlight, mainly in tropical bat species [24–27]. Flying closer to vegetation may hamper prey capture success, as background echoes may interfere with prey echo, especially for open-space and edge-space foragers [12]. The potential benefit of extra safety by flying close to the vegetation is therefore a trade-off between predator avoidance and foraging efficiency, which may be influenced by light.

In order to study the interaction between vegetation and flight behaviour, high resolution information on both flight pattern and vegetation structure is essential. Very few studies have explored the three-dimensional (3D) spatial data of vegetation to study bat behaviour. Some laboratory-based evidence demonstrates that bats use visual and auditory cues to navigate [28] and fly in stereotyped flight paths [29], but studies in real-life environments are sparse, most likely because of the labour-intensiveness of 3D flight path assessment. Few studies have studied bats in their natural habitat using remote sensing methods such as Light Detection and Ranging (LiDAR) [17, 30–33]. While aerial laser scan (ALS) was preferred to cover larger areas [17, 31–34], very fine-scale vegetation information can be obtained at the plot level with terrestrial laser scanning (TLS), especially below the canopy, where bats potentially fly to avoid predators or search for prey [35–37]. TLS has become the method of choice for precise 3D scanning of vegetation relevant for bat habitat, as it is the best approach for scanning the canopy and the vegetation below [38]. In previous studies, spatial distribution of bats was assessed using ultrasonic bat detectors, thermal imaging [39], GPS (Global Positioning System) tags [34] or mist nets [25]. While bat detectors or mist nets provide limited detail on vertical stratification of bats [11, 25], thermal imaging can mainly be used in open areas, as it is difficult to combine data from multiple cameras in dense forest and thus to reconstruct bats’ flight patterns. Several cameras are needed and must be carefully positioned with greater but precisely known distance and exact angle in order to calculate precise positions of bats, and this has to be done again every single time they are set up in a new environment. Therefore, these techniques are much less suitable to study the fine-scale species-habitat relationship of bats.

Nowadays, acoustic localisation can overcome some disadvantages of these methods. It offers great opportunities to precisely study animal movements on a fine but limited spatial scale. Acoustic localisation is done by calculating the time-of-arrival-difference (TOAD) of each signal between several microphones [40, 41]. Acoustic tracking using microphone arrays is easy to deploy: it is limited to mounting a frame with microphones at fixed positions on a tripod. As long as one knows the position and the angle of the frame relative to the ground plane, bats' positions can be calculated relative to the array [41]. As echolocating bats produce numerous echolocation calls per second, the technique allows for tracking with a high spatial and temporal resolution, and echolocation calls can be simultaneously used for species identification. The method is thereby a great complementation to GPS tracking, which provides much less precise spatial data, but at a much larger scale and specific for each individual. Another benefit of acoustic localisation is that animals do not need to be captured and carry a logger or transmitter, so their behaviour is not altered by this technique and even very small bat species can be tracked.

Here, we show that the difficulty of accurate assessment of the interaction between bat flight behaviour and spatial structures can be resolved with combining acoustic bat tracking and LiDAR vegetation scans. This opens up the possibility to acquire knowledge on the fundamental mechanisms on how bats interact with their environment. Recent developments in portability and ease of deployment of both techniques facilitate quick collection of spatial data on vegetation structure and bat flight behaviour in the field. Although it entails challenges with combining mass-volumes of fine-scale bat movements and vegetation information, here we show the feasibility and potential of combining those two methods for future studies on bats. This combined method could be applied as well to other systems linking movement patterns and measurements of 3D space.

## Methods

### Field sites

**Fig. 1:**
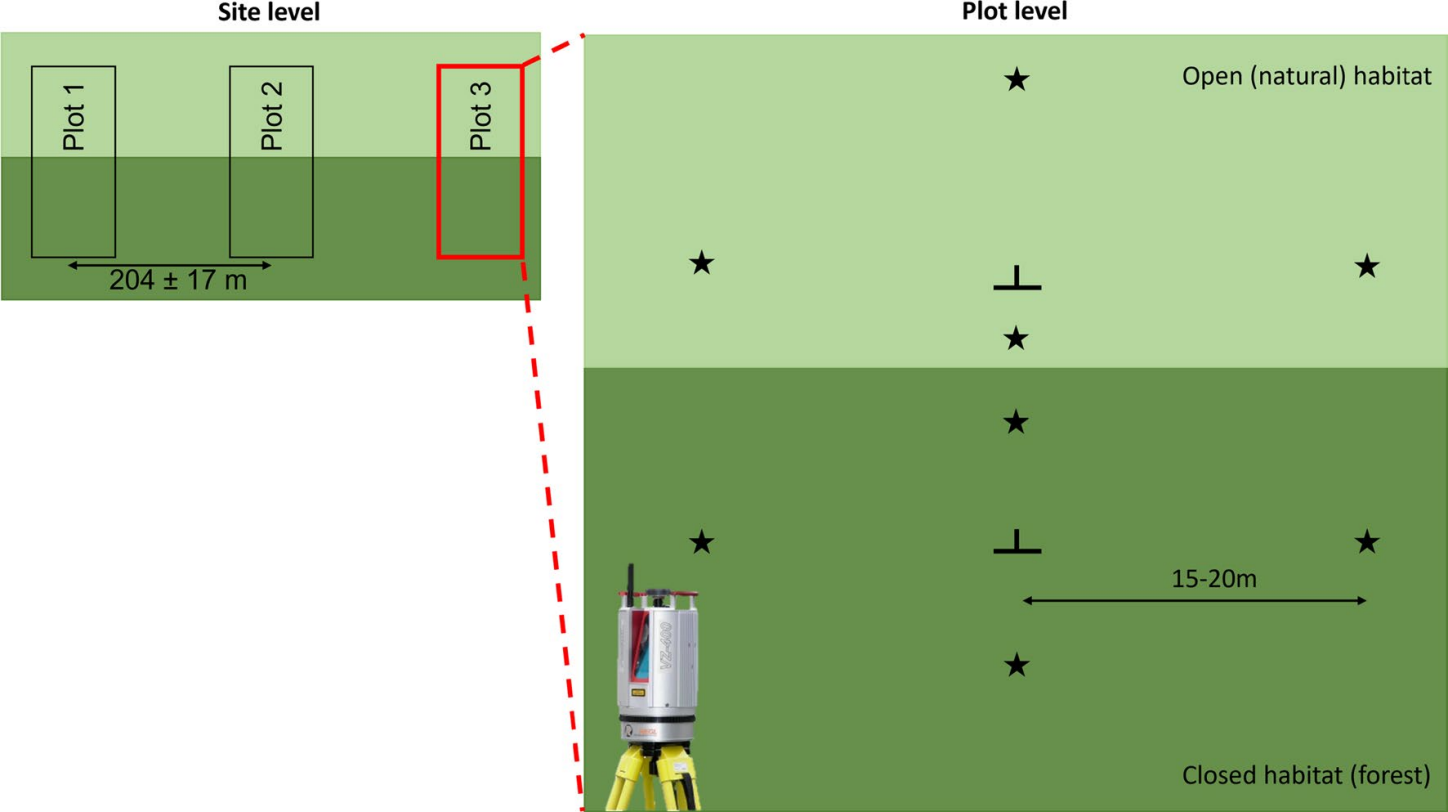
Set-up to collect LiDAR data at one plot within a site with a RIEGL VZ-400 terrestrial laser scanner. To include both open and closed vegetation structures, we deployed microphone arrays (T-shaped) at the forest edge and in the forest and we scanned the plot up to 15–20 m from the array. Each star represents an individual scan location at which both an upright and tilted scan were done

Acoustic bat tracking was combined with LiDAR scans in forest edge habitat at seven experimental sites set up to study the effect of artificial light at night on the forest-edge ecosystem [42]. The sites are located in the Netherlands and consist of either coniferous forest with Scots pine (*Pinus sylvestris*) or Douglas fir (*Pseudotsuga menziesii*), or mixed forest with Scots pine, common oak (*Quercus robur*) and birch (*Betula* sp.). At each site we collected data at three plots (Fig. 1). The distance between the centre of two plots varied between 88 and 386 m (average 204 m; standard error, s.e. 17).

### Acoustic localisation

#### Data collection

Bats were acoustically recorded from 15 min before sunset to 15 min after sunrise for a total of 27 nights between May 8th 2020 and August 9th 2020. Up to seven microphone arrays were deployed at one site per evening in the open area, at the forest edge and in the forest to account for different vegetation structures (three plots per site, two to three arrays per plot).

#### Technical description of the system

**Fig. 2:**
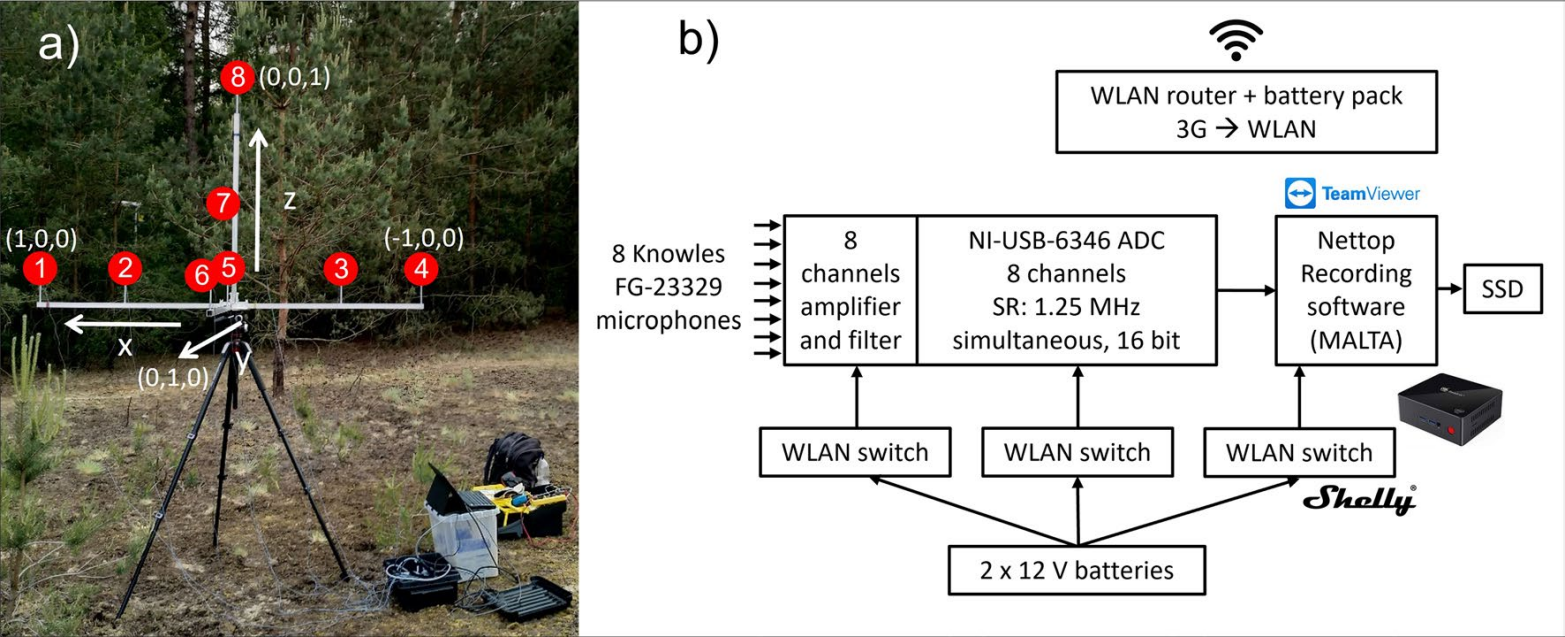
Acoustic localisation setup. **a** Array set-up in the field, red dots indicate the positions of the eight microphones (also numbered), **b** schematic of the set-up

Each array consisted of eight microphones (omnidirectional microphones FG-23329 Knowles Electronics, Itasca, IL, USA), fitted on an aluminum frame with arms in x, y and z directions (see Fig. 2a for precise layout). The array frame could be disassembled for easy transport in remote field sites only accessible by foot. The microphones were connected to a custom-made amplifier and filter unit (Fig. 2b). Sound recordings were digitised with an Analog–Digital-Converter USB-6346 (DAQ) (National Instruments, TEX, USA) at a sampling rate of 300 kHz and 16 bit resolution. MALTA Software (Microphone Array Localisation Tool for Animals, version 3.6, CAE Software & Systems, Germany) allowed real-time visualization of time series of all channels and computation of real time spectrogram of one channel at a time. All recording parameters were controlled and set in the MALTA Software, and all sound recordings were stored on Mini PCs (Gemini X, Beelink). Recording systems were remotely controlled and monitored using WLAN routers (TP-Link M7200 MiFi) and TeamViewer (TeamViewer GmbH, Germany).

Each system was battery-powered by one 12 V 20 Ah battery for the Mini PC and the DAQ and one 12 V 5 Ah battery for the amplifier, adding up to 25 Ah in total. The power requirements of the entire system were 1.3 A at 12 V, i.e. approximately 15 W, allowing for 20 h of recording. The recordings were stored on an external 1 TB SSD. All the recording equipment including batteries easily fits in a 20 L box (Additional file 1: Fig. S1).

#### Calculating 3D positions

Each echolocation call reaches each of the eight microphones at a different time because of the distance between the microphones. The time-of-arrival-difference (TOAD) between the signal of the reference microphone (in this case the top microphone) and the signal of each of the other microphones was determined by cross correlation using a custom-built software (TOADSuite, P. Stilz, J.C. Koblitz and H.R. Goerlitz) [43] in MATLAB R2020a (The MathWorks, Inc., Natick, MA, USA). The bat’s position at the moment of signal emission was calculated based on these TOADs [44].

For the analysis two approaches are possible: (1) the 3D position of every call localised with sufficient precision is considered as an individual data point. (2) subsequently, flight paths based on the spatial temporal pattern of successive localised echolocation calls can be computed. Note that the spatial coverage of the array is limited to a hemisphere with a radius of 5–20 m depending on bat species. Animals frequently leave and re-enter this hemisphere and it is impossible to determine whether the same or a different bat is recorded.

#### Localisation error assessment

Technically, only four microphones are needed for 3D localisation. In this study we added four extra microphones, resulting in an overdetermined array with eight microphones (Fig. 2). The use of an overdetermined array allows assessment of localisation error by comparing the theoretical TOADs based on isotropic spherical sound spreading from the localised sound source position with the real TOADs of the incident sound at the multiple microphone positions. Two types of localisation errors can be assessed, namely the radial error and the tangential error. The radial error defines the difference between the actual and calculated 3D position of the sound source in a direct line to the centre of the array. The tangential error defines the difference between the actual and calculated 3D position of the sound source in the plane perpendicular to the axis between the centre of the array and the calculated position (see Additional file 2).

Large localisation errors may occur for short and faint calls with short inter-pulse interval during the feeding buzz phase (the moment bats capture an insect; [20]), or when calls from two individuals are recorded simultaneously, leading to cross correlation mismatch between the microphones. As a rule of thumb, an accurate localisation can be achieved in a distance of one to ten times the array dimension (in this case 2 to 20 m with an array aperture of 2 m). 3D positions based on recordings of pulses very close to the array are the least precise localisations due to reflection and shading artefacts from the array frame, relatively large TOADs because of the array geometry and microphones receiving highly different signal shapes of different emission directions. Therefore, positions within two meters of the centre of the microphone array were excluded (Additional file 2: Fig. S4). Positions located more than 20 m away were kept, as it is possible to detect and localise very loud calls emitted in the open area. Moreover, as the aim of this method is to combine bats' positions with fine-scale vegetation data, positions were excluded if one of the two localisation errors was greater than 0.5 m.

#### Species identification

Sound files were analysed with the Tadarida software ([45], online repository: https://github.com/YvesBas, January 2021 version) to detect and classify sound events. As the identification of bats to the species level is difficult based on their echolocation parameters, we limited identification to the following bat guilds: the ENV group including *Eptesicus* spp., *Nyctalus* spp. and *Vespertilio* spp. that are open space aerial foragers, the *Myotis* group including *Myotis* spp. that forage close to and within foliage or over water surfaces, and the *Pipistrellus* group including *Pipistrellus* spp. that are edge space aerial foragers [10, 18].

Lastly, we linked each microphone array derived 3D position to the species group identified by Tadarida, using the detection time of the bat calls.

### LiDAR

Multiple returns Terrestrial LiDAR data were collected from June 2020 to April 2021 with a RIEGL VZ-400 terrestrial laser scanner (RIEGL LaserMeasurement Systems, Horn, Austria). Scans were done under leaf-on conditions (presence of foliage on deciduous trees), but plots with almost exclusively coniferous species were scanned later in the season. For each plot in which we linked acoustic localisation with LiDAR, scans were done at 7–16 locations (dependent on the number of arrays and density of the understorey vegetation), following the setup shown in Fig. 1. One scan was always done close to the microphone array, and additional scans were done at a distance of ~ 15 to 20 m from the array, to ensure that we captured the vegetation structure in the whole plot. At each scan location, two scans were done; the first scan with the scanner straight up, covering zenith angles between 30° and 130° off nadir. The second scan was acquired with the instrument tilted at 90° from the vertical to sample the full hemisphere. Scans were done with an angular resolution of 0.06 degrees, resulting in a cloud density with a mean Euclidean nearest neighbour distance of < 2 cm within the plot. Reflective targets were used to co-register and align the individual scan locations using RIEGL’s RiSCAN Pro Software version 2.8.0 [46]. Finally, the co-registration was optimised using the Multi-Station Adjustment (MSA) algorithm, within RiSCAN Pro. MSA modifies the position and orientation of individual scan locations in several iterations to calculate the best overall alignment. The resulting point clouds were filtered based on the deviation of the returned LiDAR signal. Returns with a higher pulse deviation often represent semi-returns, softer targets or noise which can hinder further analysis [47]. Therefore, all points with a pulse deviation higher than 15 were filtered out. Per plot this resulted in point clouds containing between 17 and 206 million points, depending on the size of the area scanned and the vegetation density.

### Combining LiDAR and acoustic localisation

Microphone arrays were set up during the LiDAR scans in exactly the same position and angle as when tracking bats (Additional file 3). The array is thus present in both 3D datasets. The array coordinates (the four ends and the centre of the frame) in LiDAR scans were subsequently used as a reference to apply a rigid body transformation (translation and rotation, [see Additional file 3]) on bats’ positions to align them with the coordinate system of the vegetation scans in CloudCompare (version 2.12 beta, 2022).

Distance to the vegetation can be directly assessed by calculating the distance for each bat’s position to the closest vegetation point using the Cloud-to-Cloud Distance computation tool in CloudCompare. However, isolated vegetation points may interfere with the calculation. Therefore, the point clouds are ‘voxelised’ by converting data into a 3D volume of data values. This yields a 3D grid with the number of points per voxel indicating voxel specific vegetation density [48]. Unlike point cloud data, voxels have a defined length, width, area and volume, which can contain quantitative information on the space occupied by vegetation. These parameters depend on voxel size; small voxels result in data redundancy, while large voxels overestimate the space occupied by objects [48, 49]. According to Ross et al. [49], a 10–25 cm resolution is the optimal size for estimating canopy gaps in forest plots, which is an important factor to take into account when studying bat flight behaviour. This also corresponds to the closest distance between target and background clutter required for bats such as *Myotis nattereri* to detect prey [21]. Lasvoxel tool in LAStools (version 210418, rapidlasso GmbH, Gilching, Germany) was used to build a 3D grid of 20 cm^3^ cubic voxels. For each voxel we thus obtain an occurrence value of vegetation points, and we can define voxels with a vegetation count value below a specific threshold as background voxels.

## Results

### Overall activity

Out of 138 recorded array nights (one array night is a full night of recording for one microphone array), 6822 (s.e. 1465) 3D positions per night were calculated in average. By defining a bat pass as a 10 s file containing at least one position, this resulted in 326 (s.e. 38.3) passes per night in average. 93.52% of the 3D positions were assigned to the *Pipistrellus* group, 6.29% to the ENV group (*Eptesicus* spp., *Nyctalus* spp. and *Vespertilio* spp.) and 0.19% to the *Myotis* group. This is consistent with previous studies carried out at the same sites using different acoustic monitoring devices [50, 51].

### Individual tracks

**Fig. 3:**
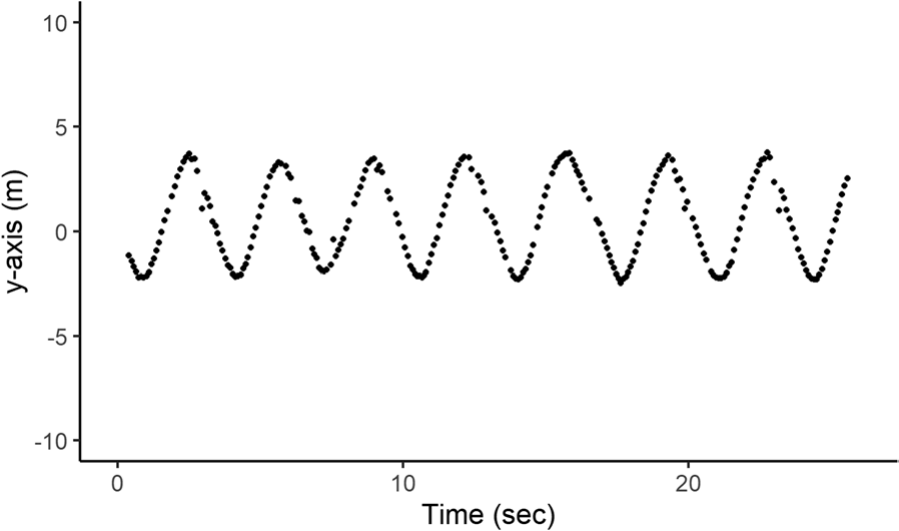
Individual flight track of a bat in one dimension (Y coordinate over time). Y = 0 m corresponds to the centre of the microphone array (positions are more than 2 m away from the array centre in three-dimensional space)

**Fig. 4:**
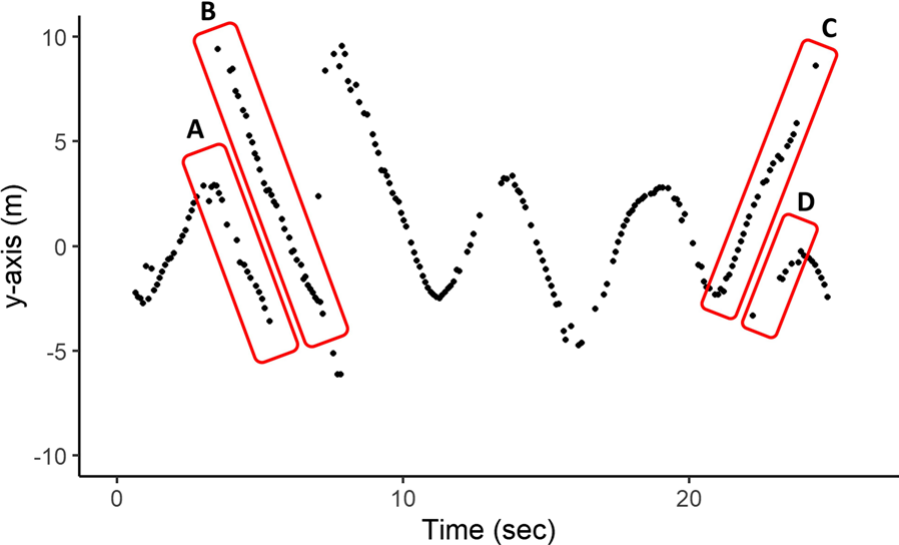
Multiple flight tracks of bats in one dimension (Y coordinate over time). Y = 0 m corresponds to the centre of the microphone array (positions are more than 2 m away from the array centre in three-dimensional space). Red boxes highlight the presence of two individuals at the same time. A and B correspond to two individuals with distinctive flight tracks. C and D are either tracks of the same individuals or from two other individuals

Individual tracks can be constructed using subsequent 3D positions, which has the advantage that two individuals recorded at the same time can be spatially separated. This can be done in some cases using just one spatial dimension (i.e. x, y or z coordinate values; see Figs. 3, 4). In Fig. 3, one bat flies back and forth from 4 m at one side of the array to 2 m at the other side of the array with a very regular movement pattern (see Additional file 1: Fig. S2 for the track in 3D). On the other hand, Fig. 4 shows two to four distinctive flight tracks. As individuals leave and enter the recorded hemisphere, it is however impossible to assess whether the same or different bats are recorded over time. The maximal recorded distances for the ENV, *Pipistrellus* and *Myotis* groups are respectively 37.25 m, 34.27 m and 20.70 m from the array.

### Integration of LIDAR and acoustic localisation

#### Case study 1: Stereotyped flight paths

**Fig. 5:**
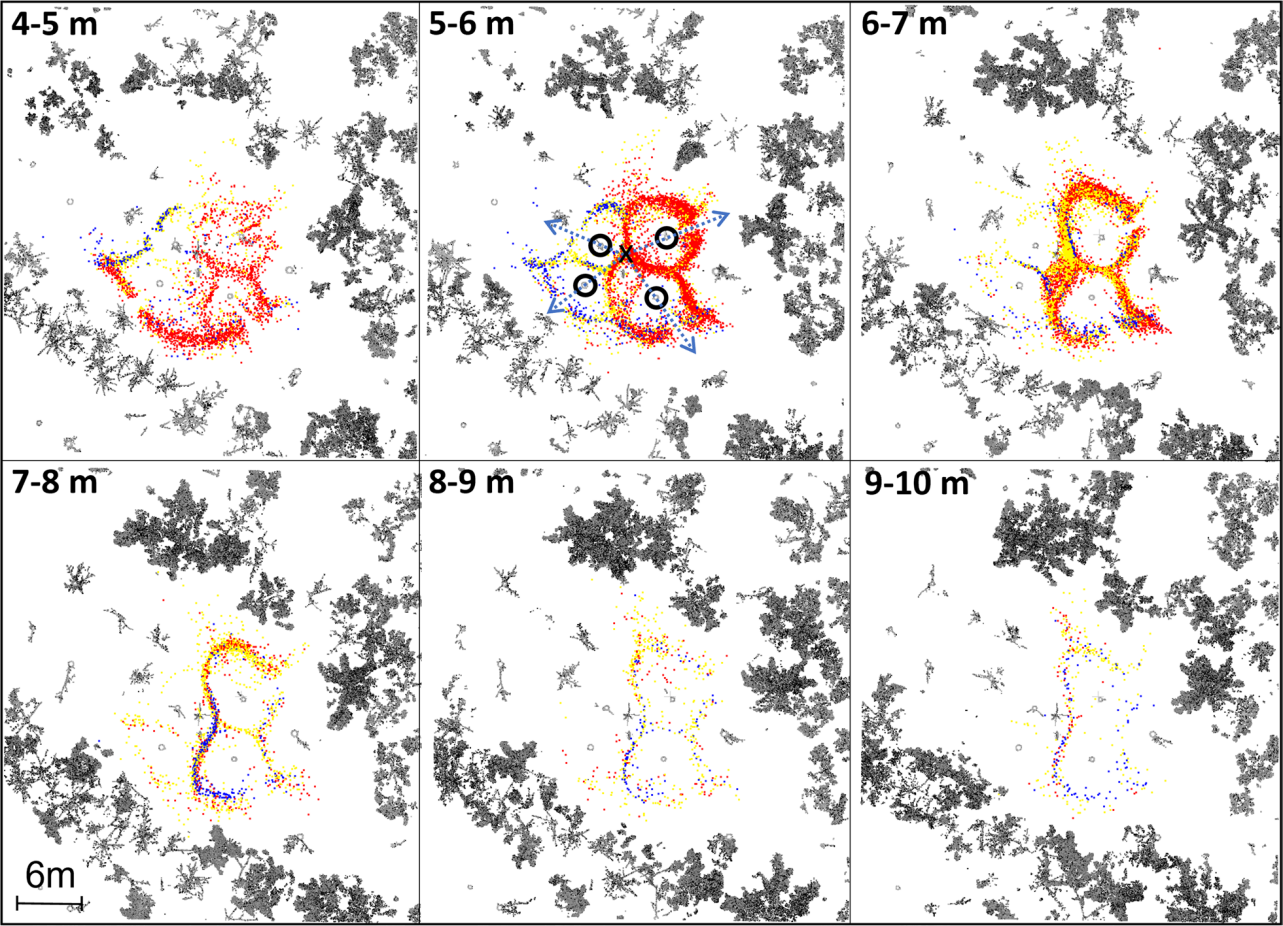
Top view of *Pipistrellus* spp. positions (coloured dots) integrated with a vegetation scan (black dots) at heights that included most of the calculated bats’ positions (from 4 to 10 m above the ground). Each colour corresponds to a different recorded night (blue for June 9th 2020, red for July 28th 2020 and yellow for July 30th 2020). In the second plot (from 5 to 6 m high), the cross indicates the microphone array’s position. The tree trunks (circled) produce acoustic shadows beyond them (represented by the blue arrows)

Spatial alignment of vegetation scans and bats’ positions shows that bats’ positions can be well aligned with the vegetation, as represented in Fig. 5. Obstacles like tree trunks obstruct sound propagation of bat calls when in between the bat and the microphone array, thus the echolocation calls cannot reach the microphones and cannot be localised. These acoustic shadows validate the alignment of the two 3D datasets by matching the missing bats’ positions with the obstacles in LiDAR scans. Figure 5 shows data from three nights of recordings at the same plot. Pipistrelles use stereotyped flight paths in cluttered environment by circling around the trees each night in each vegetation layer (subfigures of Fig. 5). However, it is not feasible to estimate how many individuals were flying in these stereotyped flight paths based on acoustic recordings. This first case study is one of the few pieces of evidence of stereotyped flight paths of pipistrelles in their natural habitat [52].

#### Case study 2: Distance to vegetation

**Fig. 6:**
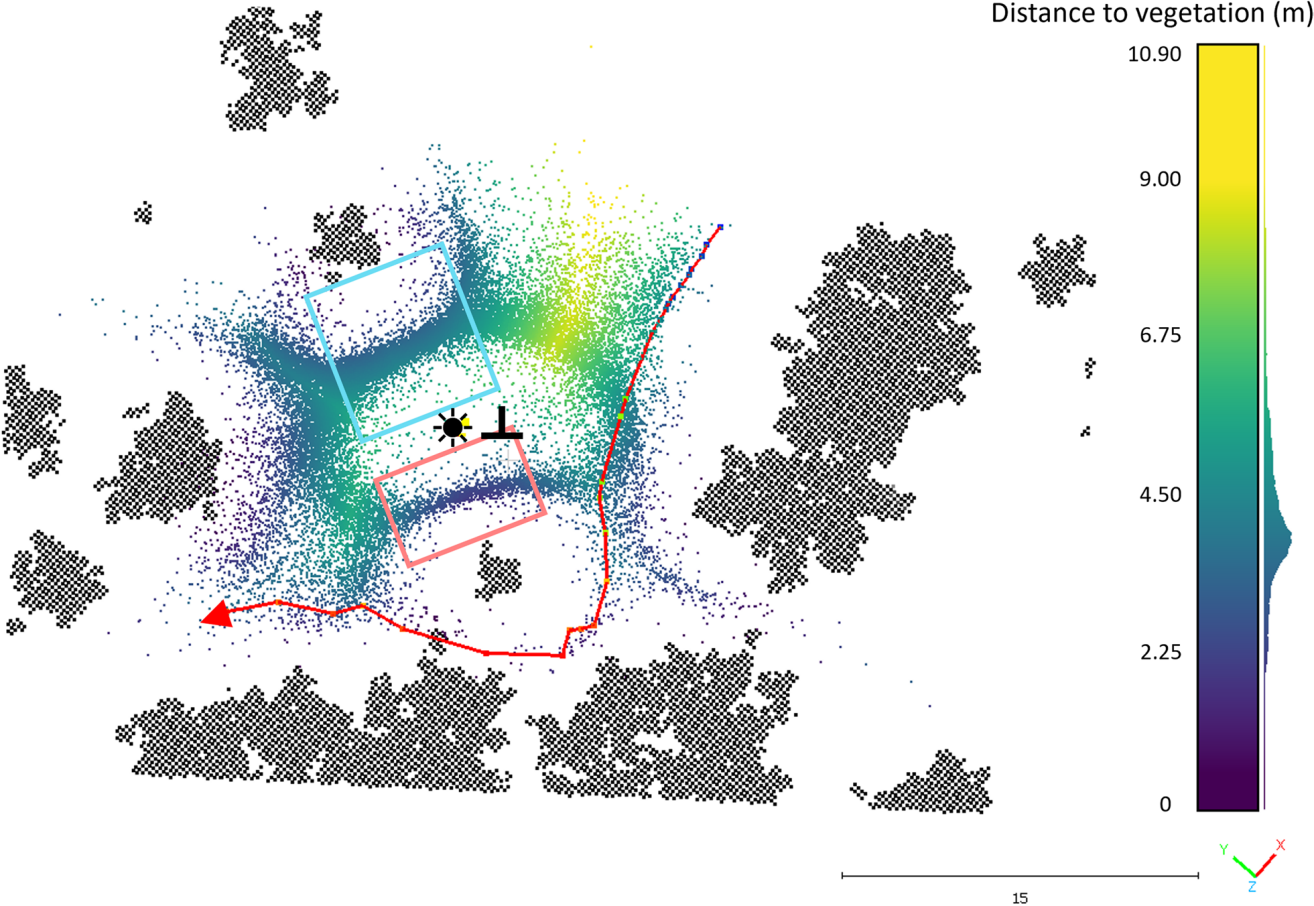
Distance of bat activity relative to the vegetation. Top view of *Pipistrellus* spp. positions (coloured dots) integrated with the voxelised vegetation scan (black dots) within the horizontal plane between 3.5 and 4.5 m above the ground. The T-shape represents the array. The red arrow shows an example of a flight trajectory. Voxels have a size of 20 × 20 × 20 cm. Only voxels with at least ten vegetation points are marked as ‘vegetation voxels’ as this preserves a fine-scale resolution in vegetation while filtering out background voxels containing isolated vegetation points. The distance to the vegetation corresponds to the absolute distance (in meters) of each bat position to the closest vegetation voxel containing at least ten vegetation points. The black icon next to the microphone array shows the position of the lamppost (height of 4 m), which is part of the experimental setup of the “Light on Nature” sites. The highlighted sections indicate the narrow (light red) and the wide (light blue) corridors described in Fig. 7

**Fig. 7:**
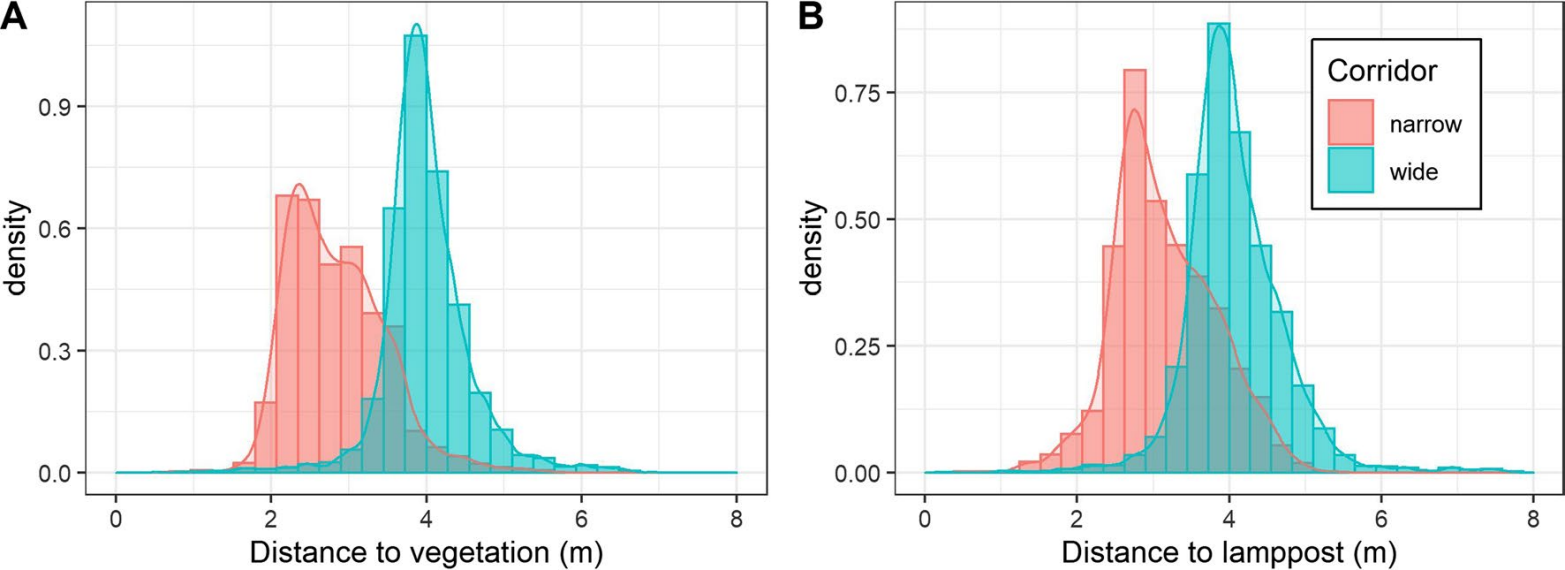
Space use of pipistrelles in response to artificial light in a narrow (4.8 m wide, n = 1342 positions) and a wide (7.6 m wide, n = 6463 positions) corridor shown in Fig. 6. **A** Distribution of bats’ distance to the closest vegetation voxel containing at least ten vegetation points. **B** Distribution of bats’ distance to the lamppost

The second case study describes the distance that bats keep to the vegetation structure and obstacles. Figure 6 depicts the distance pipistrelles keep to the vegetation in forest-edge habitat. The flight path of one individual along the forest edge is also reconstructed as an example (Fig. 6). Spatial data are structured in 20 × 20 × 20 cm voxels. In this case study, pipistrelles fly around a lamppost, while keeping their distance both from the vegetation and the lamppost, as shown in Figs. 6 and 7. When flying in a wider corridor, bats stay further away both from the vegetation (Fig. 7a) and the light source (Fig. 7b). In the wide corridor (7.6 m wide), bats fly in average at 4.07 m from the lamppost and 3.99 m from the vegetation (Welch t-test = 7.06, *p* < 0.001). In the narrow corridor (4.8 m wide), bats fly in average at 3.14 m from the lamppost and 2.84 m from the vegetation (Welch t-test = 12.4, *p* < 0.001). Thus, pipistrelles fly closer to the vegetation than the lamppost, but they also keep a certain distance to the vegetation to avoid clutter.

**Fig. 8:**
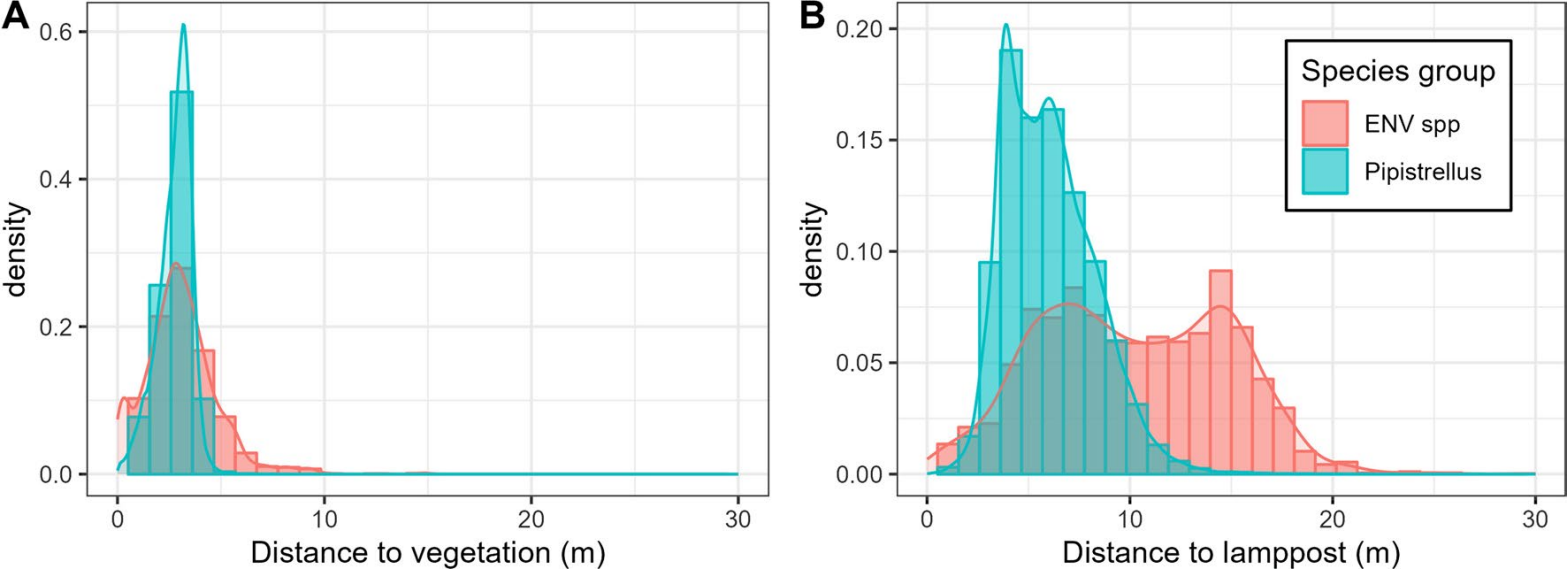
Space use of the ENV group (n = 1790 positions) and the *Pipistrellus* group (n = 62,336 positions) in forest edge habitat. **A** Distribution of bats’ distance to the closest vegetation voxel containing at least ten vegetation points. **B** Distribution of bats’ distance to the lamppost

Myotis and ENV groups data from the same plot are available in Additional file 1: Fig. S3. Pipistrelles and ENV groups exhibit a different use of space (Myotis group was not compared, as only two tracks were recorded, see Fig. S3c). While most pipistrelles fly up to 4 to 5 m from the vegetation, ENV species have a wider distribution of distance to the vegetation (Fig. 8a; the two distributions are significantly different according to the Kolmogorov–Smirnov test, D = 0.22, *p* < 0.001). Moreover, pipistrelles generally fly closer to the lamppost than ENV species (Fig. 8b; the two distributions are significantly different according to the Kolmogorov–Smirnov test, D = 0.46, *p* < 0.001).

## Discussion

In this study, we show that the combination of acoustic localisation and LiDAR vegetation scanning is a method of great potential to study the interaction of bats with their immediate surroundings. The ability to study bat behaviour in relation to fine-scale structures is of relevance as many bat species strongly rely on these for nightly foraging and commuting routines [53–55]. They may change this interaction depending on momentary and local weather [56], light conditions [57] or prey availability [36, 37]. By combining acoustic localisation and LiDAR, these interactions can be precisely quantified [58]. With the improvements on the acoustic tracking system, microphone arrays have become easy to deploy and recordings can now be remotely controlled and monitored for two full nights. The ability to use the microphone array system remotely controlled for consecutive nights allows for additional assessment of temporal changes in addition to spatial data.

Acoustic tracking data have a very high spatial accuracy, with spatial resolution of few centimetres. This is very valuable to study the fine-scale interaction of bat flight with habitat structure, which would not be possible with GPS or radio tags. It also provides very high temporal resolution data, as bats emit numerous echolocation calls per second. As we used an overdetermined array, it is possible to use differences in predicted and observed TOADs to estimate localisation errors.

The limitations of acoustic localisation depend on a complex relation of a multitude of parameters, for example shading artefacts from the array frame, array geometry, call directionality and shape, or inter-pulse intervals. Interfering calls and low signal-to-noise ratio can limit the localisation precision. Therefore, the selection of an optimal array setup depends on the task to solve and the recording conditions. More accurate localisation can be achieved with larger TOADs using of an array with a larger aperture. However, if the targeted sound is highly directional, it may not reach some of the microphones. Here, an array with a smaller aperture may remain a better option for good localisation results.

The range for spatial detection is species-dependent, as acoustic parameters of echolocation pulses vary across bat species [59]. ENV calls are often louder and at lower frequencies, and therefore can be detected and localised further away. *Myotis* species tend to reduce their call amplitude when flying in cluttered environment and approaching prey [60], thus they will be less detectable. However, the error variance in 3D positions calculation follows the same patterns between the three species groups (Additional file 2: Fig. S4).

Combining 3D data obtained by the two different techniques requires precise alignment, but we show this is well feasible with the use of reference locations, in our case the array position itself (but if necessary, more reference locations can be added). As shown in the first case study, hard objects, such as tree trunks, create acoustic shadows and impair the ability to localise bats when in between the bat and the microphone array. Although this can be solved with the construction of flight paths, LiDAR data may further be helpful to solve potential issues with acoustic shadows produced by obstacles as these data reveal the location of such obstacles. Strictly speaking, the LiDAR data do not validate the 3D bats’ positions, but precisely explain the acoustic shadows produced by trees in case of proper alignment.

This first case study is also one of the few pieces of evidence of stereotyped flight paths of pipistrelles in their natural habitat [52]. As already suggested by Hulgard et al. (2016) [29] in the big brown bat (*Eptesicus fuscus*), pipistrelles may also reduce their sensory processing load for navigation in known area in order to ameliorate their foraging efficiency. Combining the bats’ positions with vegetation scans provides an additional layer of information in our understanding of the acoustic field of view in echolocating bats. The ability of prey detection depends on the distance to clutter of a foraging bat. Therefore, it is highly relevant to get high-detailed information on vegetation through LiDAR scans and combine this with acoustic localisation data to study how vegetation affects echolocation behaviour of bats in cluttered habitat. This would allow to investigate the plasticity in echolocation signals at a fine-scale spatial resolution in the field.

The second case study shows the potential of unravelling the interaction of bat flight behaviour and the vegetation structure. Different bat guilds keep distinct distances to obstacles; in our example, open-space foragers such as ENV species stay further away from the vegetation and from a lamppost than opportunistic species like pipistrelles. Therefore, measuring the distance of bats to the vegetation is particularly relevant to understand niche segregation of bat guilds in relation to the density of habitat clutter.

How bats respond to abiotic factors, such ambient light by the moon or artificial light sources and how these interact with vegetation [50] can be studied in much more detail using acoustic tracking and LiDAR. The deterrent effect of light could also be studied in greater detail by mapping the light level around lampposts and link this with the vegetation structure around the light source and bat 3D activity.

This example also shows the potential of this method to precisely look at behaviours such as the use of corridors by bats and their flight characteristics via the analysis of their trajectories, which has important implications for bat protection measures at the landscape level. Lastly, parameters such as flight speed or straightness of the flight trajectories can also be computed to evaluate bat responses to obstacles such as vegetation.

Bats are appropriate model organisms to validate our combined method, as their flight behaviour is shaped by habitat characteristics and their echolocation signals are excellent for high-resolution acoustic tracking. However, combining LiDAR with acoustic tracking could be applied for other vocalizing organisms (i.e., nocturnal species for which visual survey methods are ineffective such as crickets, katydids [61] and frogs [62, 63]). Combining the two techniques could also help to better understand acoustic behavioural changes in shrews in response to habitat clutter [64], or map song posts and territories of songbirds [65–67]. The main criterion to combine LiDAR with acoustic tracking is to use one or more common objects in both datasets (in our case, the array) as reference for co-registration. At least three reference points (here we used five points) are needed to apply a rotation and translation matrix on one 3D dataset to align it with the other one. LiDAR could also help to precisely map microphones that are separated from each other on larger distances and synchronized by radio-transmission or GPS signal in thick vegetation.

## Conclusions

Combining techniques as acoustic localisation and LiDAR allows to precisely map bat flight movements in response to spatial structure, opening up the possibility to address open and novel questions on fine-scale bat behaviour, such as niche segregation between different bat guilds, and responses to artificial light at night. While it is important to consider wider landscape composition to study forest management and bat conservation, studies on the local, fine scale may prove highly important to provide bats high-quality foraging habitat. There are other vocalizing animal species, such as songbirds, of which novel information can be collected to exploit the potential of the combination of the methods, making the methodology outlined in this paper relevant for a wide range of study systems.

## Supplementary Information

**Additional file 1: Fig. S1.** Recording equipment for acoustic localisation of bats. **Figure S2.** Individual flight track of bat, top view. The dot size and the colour scale indicate respectively the bat’s height and the time of the call emission. The black cross shows the array’s position. The black dot indicates the position of the lamppost which is part of the experimental setup of the “Light on Nature” sites. **Figure S3.** Top view of bat positionsintegrated to vegetation scan. a) Pipistrellus spp., b) ENV groupand c) Myotis spp.. The T-shape represents the array. The brown dot next to the microphone array shows the position of the lamppost, which is part of the experimental setup of the “Light on Nature” sites.

**Additional file 2.** Localisation error assessment. **Figure S4:** Errors assessment over the distance to the array for positions calculated over one night of recording with substantial bat activity. A) Max TOAD distance error. B) Radial error. C) Tangential error. The red dots indicate the positions within two meters of the centre of the microphone array, where localisation can generally not be achieved precisely.

**Additional file 3.** Procedure to align two 3D datasets.

## Data Availability

All data used in this study are available from the Dataverse Digital Depository, and can be accessed on request via the link: https://doi.org/10.34894/CHW0HB.

## Abbreviations

ENV group: Group including *Eptesicus* spp., *Nyctalus* spp. and *Vespertilio* spp.
GPS: Global Positioning System
LiDAR: Light Detection and Ranging
TOAD: Time-of-arrival-difference
MSA: Multi-Station Adjustment
3D: Three-dimensional
