# Supplementary material for: Combining acoustic tracking and LiDAR to study bat flight behaviour in three-dimensional space
Source: Mov Ecol. 2023 Apr 26;11:25. doi: 10.1186/s40462-023-00387-0 (PMC10131301; doi:10.1186/s40462-023-00387-0)
Supplement: Supplementary file 1 — Additional file 1: Fig. S1. Recording equipment for acoustic localisation of bats. Figure S2. Individual flight track of bat, top view. The dot size and the colour scale indicate respectively the bat’s height and the time of the call emission. The black cross shows the array’s position. The black dot indicates the position of the lamppost which is part of the experimental setup of the “Light on Nature” sites. Figure S3. Top view of bat positionsintegrated to vegetation scan. a) Pipistrellus spp., b) ENV groupand c) Myotis spp.. The T-shape represents the array. The brown dot next to the microphone array shows the position of the lamppost, which is part of the experimental setup of the “Light on Nature” sites. [file 40462_2023_387_MOESM1_ESM.pdf]

Additional file 1

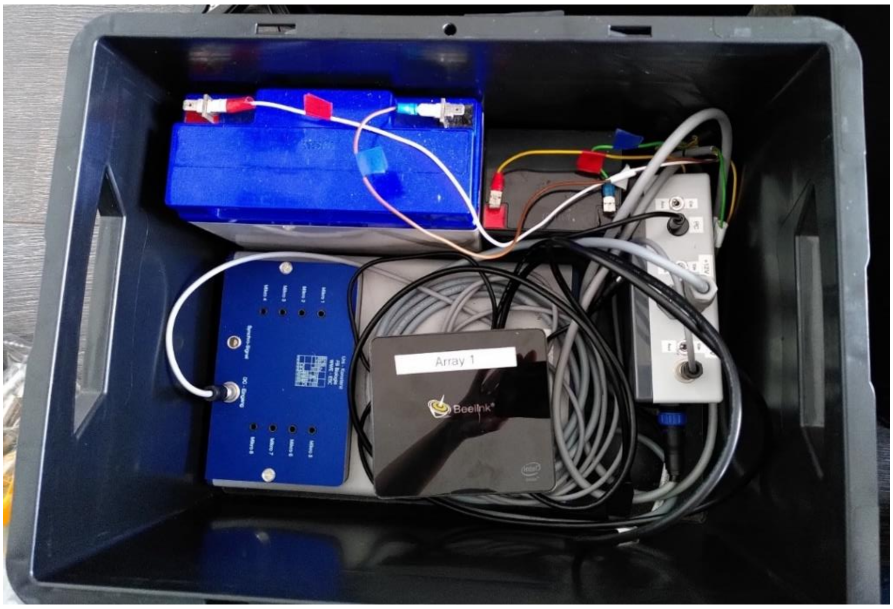

Figure S1: Recording equipment for acoustic localisation of bats.

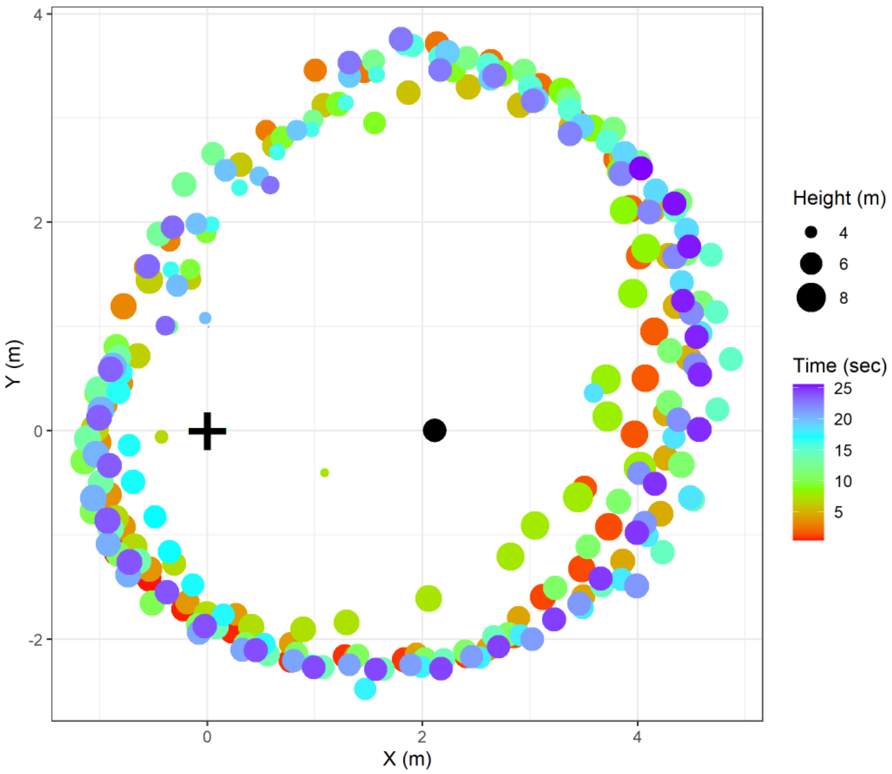

Figure S2: Individual flight track of bat, top view. The dot size and the colour scale indicate respectively the bat's height and the time of the call emission. The black cross shows the array's position. The black dot indicates the position of a lamppost, which is part of the experimental setup of the "Light on Nature" sites.

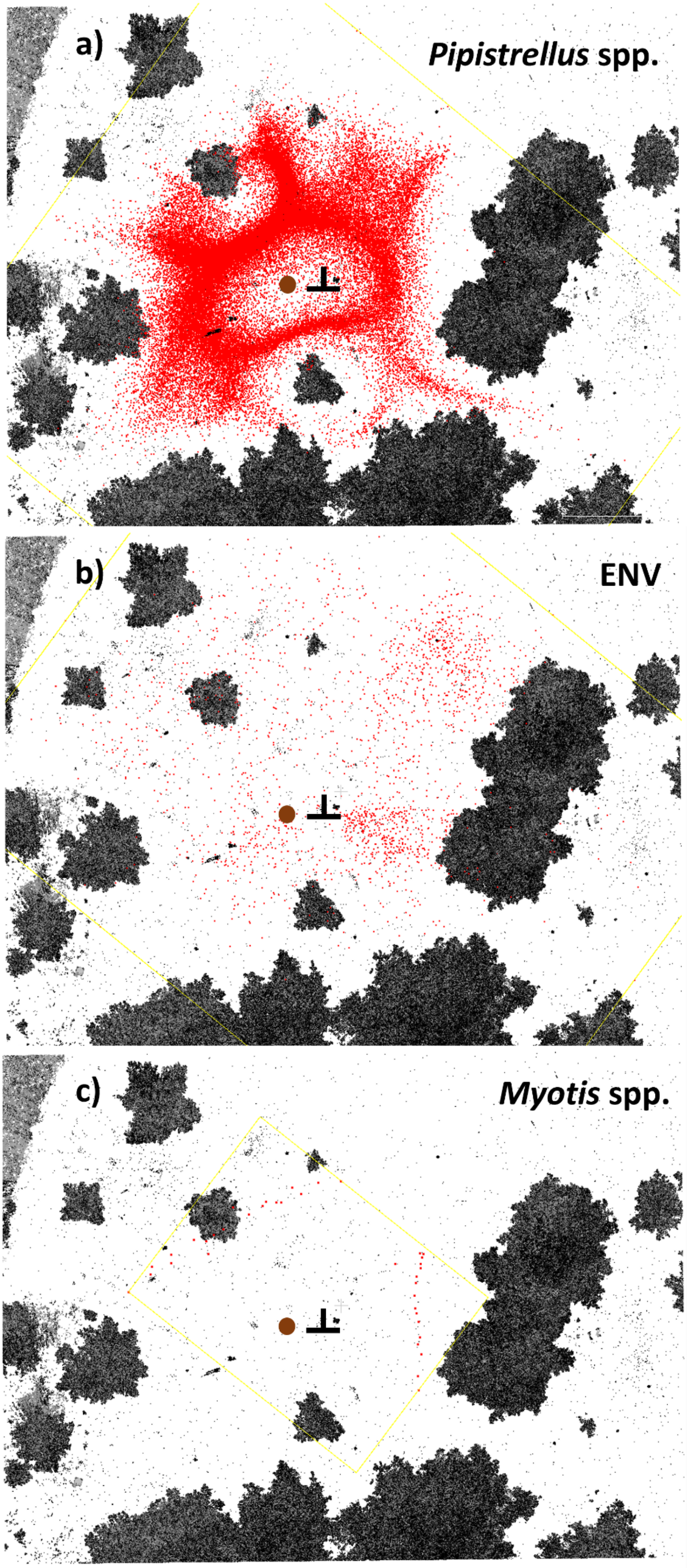
a)
Pipistrellus spp.
b)
ENV
c)
Myotis spp.

Figure S3: Top view of bat positions (red dots) integrated to vegetation scan (raw data). a) *Pipistrellus* spp., b) ENV group (*Eptesicus* spp., *Nyctalus* spp. and *Vespertilio* spp.) and c) Myotis spp.. The T-shape represents the array. The brown dot next to the microphone array shows the position of a lamppost (height of 4 m), which is part of the experimental setup of the "Light on Nature" sites.
