# Supplementary material for: Combining acoustic tracking and LiDAR to study bat flight behaviour in three-dimensional space
Source: Mov Ecol. 2023 Apr 26;11:25. doi: 10.1186/s40462-023-00387-0 (PMC10131301; doi:10.1186/s40462-023-00387-0)
Supplement: Supplementary file 2 — Additional file 2. Localisation error assessment. Figure S4: Errors assessment over the distance to the array for positions calculated over one night of recording with substantial bat activity. A) Max TOAD distance error. B) Radial error. C) Tangential error. The red dots indicate the positions within two meters of the centre of the microphone array, where localisation can generally not be achieved precisely. [file 40462_2023_387_MOESM2_ESM.pdf]

# Additional file 2

**Localisation error assessment**

Technically, only four microphones are needed for 3D localisation. In this study the array has four extra microphones, resulting in an overdetermined array with eight microphones.

The use of an overdetermined array allows assessment of the localisation error: based on the arrival time at all microphones, the location of the sound source is calculated such that the sum of all squared differences between recorded and predicted time of arrival at each microphone has the lowest value. Subsequently, the largest difference measured between predicted and recorded arrival time of a sound pulse at one of the eight microphones is defined as the "maximum TOAD error" (TOAD = Time-of-arrival-difference). This error is multiplied with the speed of sound to obtain the corresponding distance error, defined as “maximum TOAD distance error”.

Using the maximum TOAD distance error, two types of localization errors can be estimated, namely the radial and the tangential error. The radial error defines the difference between the actual and calculated location of the sound source in a direct line to the centre of the array. The tangential error defines the difference between the actual and calculated location of the sound source in the plane perpendicular to the axis between the centre of the array and the calculated location. The location reconstruction error in radial direction is approximated by:

$$radial\ error = 2 \times max\ TOAD\ distance\ error \times \left(\frac{source\ distance}{array\ aperture}\right)^2$$

On the other hand, the position reconstruction error in tangential direction is approximated by:

$$tangential\ error = max\ TOAD\ distance\ error \times \frac{source\ distance}{array\ aperture}$$

As a rule of thumb, an accurate localisation can generally be achieved within a distance of one to ten times the array aperture (in this case 2 to 20 m with an array aperture of 2 m). Figure S4 shows indeed that within 2 m of the array, the three types of errors increase drastically. Therefore, we excluded positions within two meters of the centre of the microphone array.

As the aim here is to combine bat positions with fine-scale vegetation data, we excluded positions if one of the two localisation errors was greater than 0.5 m.

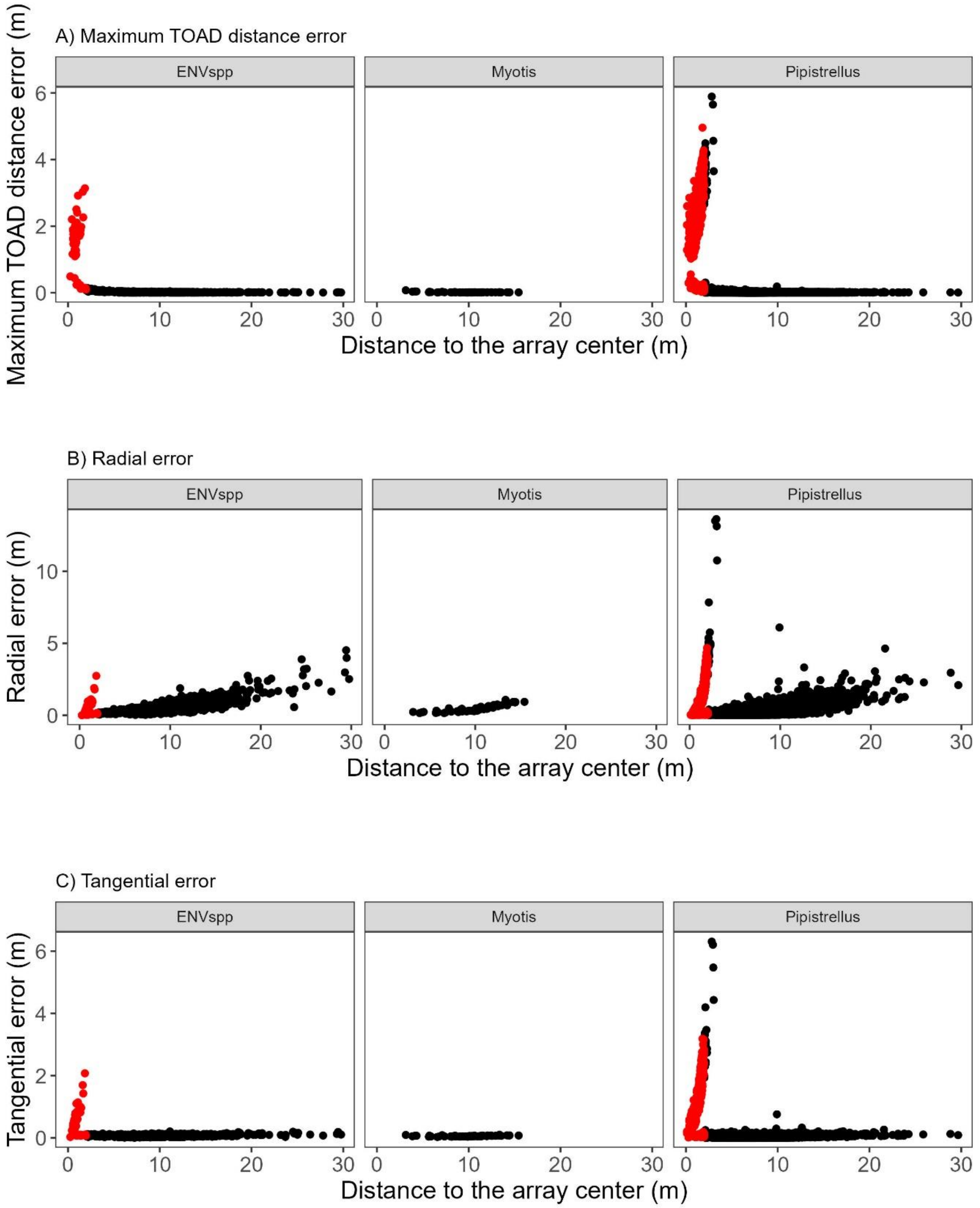


**Figure S4: Errors assessment over the distance to the array for positions calculated over one night of recording with substantial bat activity. A) Max TOAD distance error. B) Radial error. C) Tangential error. The red dots indicate the positions within two meters of the centre of the microphone array, where localisation can generally not be achieved precisely.**
