# Supplementary material for: Combining acoustic tracking and LiDAR to study bat flight behaviour in three-dimensional space
Source: Mov Ecol. 2023 Apr 26;11:25. doi: 10.1186/s40462-023-00387-0 (PMC10131301; doi:10.1186/s40462-023-00387-0)
Supplement: Supplementary file 3 — Additional file 3. Procedure to align two 3D datasets. [file 40462_2023_387_MOESM3_ESM.pdf]

Additional file 3

Procedure to align two 3D datasets (bats' positions and vegetation scan)

1) We placed two metal tubes in the ground in which we could place poles (see the picture below, with one of the metal tubes visible on the right side of the picture). We placed them such that they were aligned just behind microphones #1 and #4. Poles were removed before the start of the array recordings to avoid sound reflection or obstruction, and placed back to use later for the microphone setup at the start of the LiDAR scanning. These poles allow us to keep exactly the same array position between nights with the same angle as well, which is essential to properly combine this with the LiDAR data.

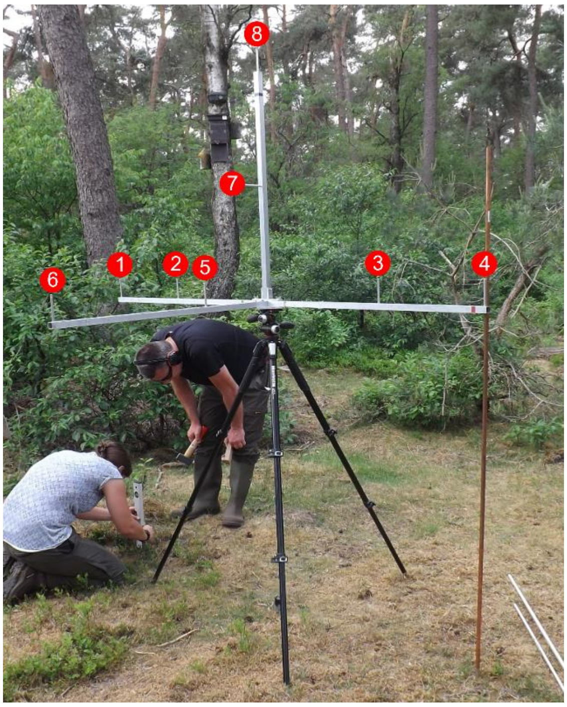

2) Create a text file with array coordinates:

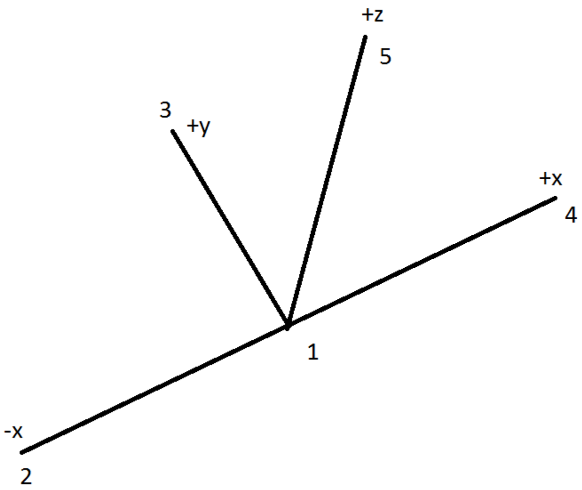

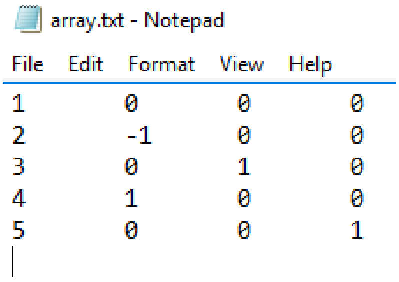
array.txt - Notepad
File Edit Format View Help

```
1	0	0	0
2	-1	0	0
3	0	1	0
4	1	0	0
5	0	0	1
```

3) Import it in CloudCompare

4) Import the LiDAR scan – Do not apply any shift on x,y,z coordinates

5) Create a cross section to localise the array

6) Select both the array data and the cross section data and click on "Aligns two clouds by picking (at least 4) equivalent point pairs

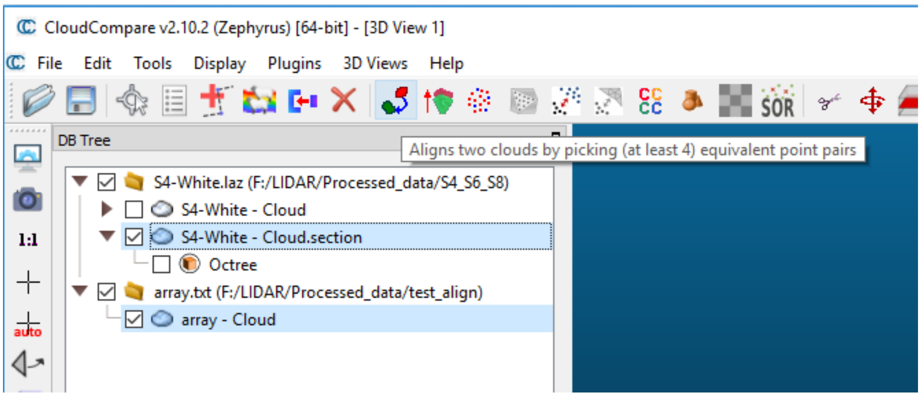

7) Use the cross section as the Reference

8) Write down the coordinates of the first array's point from the text file, then select the same point on the scan. Do the same with all the 5 points.

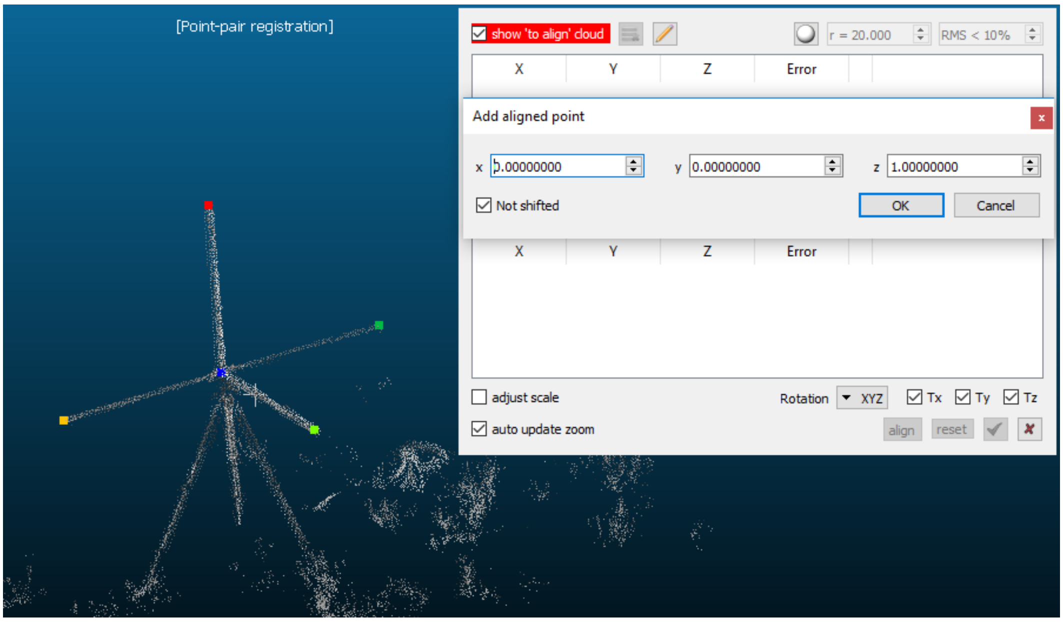

9) In the console, copy the RMS and the Rotation matrix and save it to align bats positions to TLS.

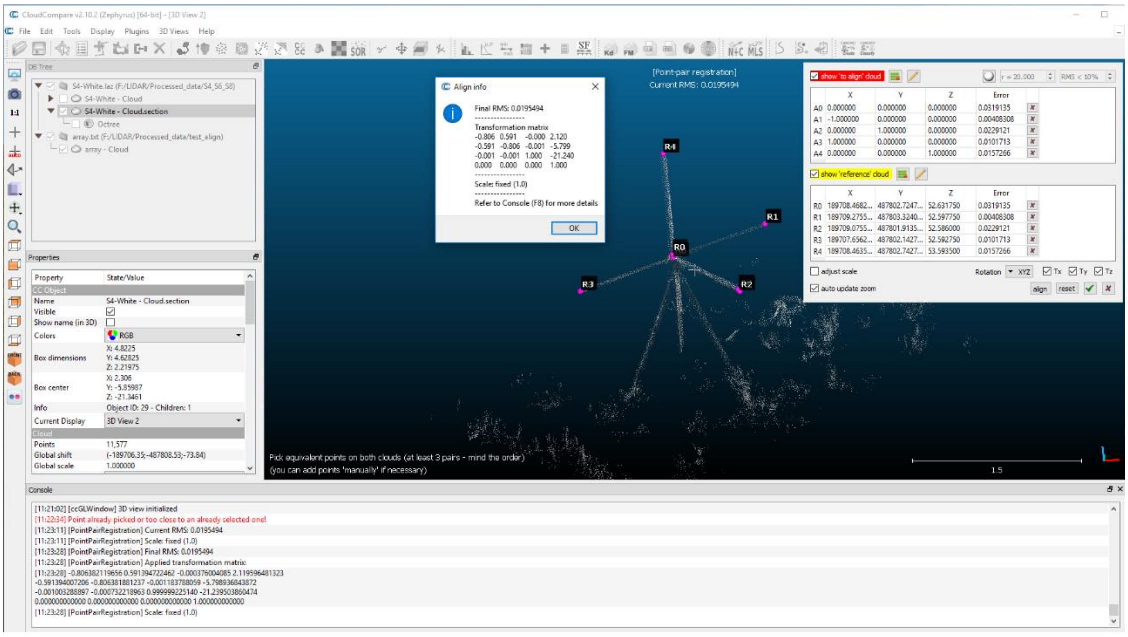

In CloudCompare, import bats positions. Select the bats cloud – Edit – Apply transformation – Copy the rotation matrix

$$\begin{bmatrix} Xnew \\ Ynew \\ Znew \\ 1 \end{bmatrix} = \begin{bmatrix} 0.598 & 0.802 & 0.010 & 0.794 \\ -0.802 & 0.598 & -0.001 & -6.566 \\ -0.006 & -0.008 & 1.000 & -16.219 \\ 0 & 0 & 0 & 1 \end{bmatrix} \mathrm{x} \begin{bmatrix} Xraw \\ Yraw \\ Zraw \\ 1 \end{bmatrix}$$

Equation 1: Rigid body transformation (translation and rotation) applied on raw data of 3D bats positions to align them with the corresponding LiDAR scan. Data are shown in Figure 5.

$$\begin{bmatrix} Xnew \\ Ynew \\ Znew \\ 1 \end{bmatrix} = \begin{bmatrix} 0.570 & 0.821 & 0.005 & 2.959 \\ -0.821 & 0.570 & 0.013 & 12.628 \\ 0.014 & -0.004 & 1.000 & -19.132 \\ 0 & 0 & 0 & 1 \end{bmatrix} \mathrm{x} \begin{bmatrix} Xraw \\ Yraw \\ Zraw \\ 1 \end{bmatrix}$$

Equation 2: Rigid body transformation (translation and rotation) applied on raw data of 3D bats positions to align them with the corresponding LiDAR scan. Data are shown in Figure 6.
